# Delineating the role of osteoprotegerin as a marker of breast cancer risk among women with a *BRCA1* mutation

**DOI:** 10.1186/s13053-022-00223-3

**Published:** 2022-04-13

**Authors:** Sarah Sohyun Park, Aleksandra Uzelac, Joanne Kotsopoulos

**Affiliations:** 1 Department of Nutritional Sciences, University of Toronto, Toronto, Ontario Canada; 2 Women’s College Research Institute, Women’s College Hospital, Toronto, Ontario Canada; 3 Department of Pharmacology and Toxicology, University of Toronto, Toronto, Canada; 4 Dalla Lana School of Public Health, University of Toronto, Toronto, Ontario Canada

**Keywords:** *BRCA1*, Osteoprotegerin, RANK, Breast cancer, Prevention, Risk prediction

## Abstract

Women with a pathogenic germline mutation in the *BRCA1* gene face a very high lifetime risk of developing breast cancer, estimated at 72% by age 80. Prophylactic bilateral mastectomy is the only effective way to lower their risk; however, most women with a mutation opt for intensive screening with annual MRI and mammography. Given that the *BRCA1* gene was identified over 20 years ago, there is a need to identify a novel non-surgical approach to hereditary breast cancer prevention. Here, we provide a review of the emerging preclinical and epidemiologic evidence implicating the dysregulation of progesterone-mediated receptor activator of nuclear factor κB (RANK) signaling in the pathogenesis of *BRCA1*-associated breast cancer. Experimental studies have demonstrated that RANK inhibition suppresses *Brca1-*mammary tumorigenesis, suggesting a potential target for prevention. Data from studies conducted among women with a *BRCA1* mutation further support this pathway in *BRCA1*-associated breast cancer development. Progesterone-containing (but not estrogen-alone) hormone replacement therapy is associated with an increased risk of breast cancer in women with a *BRCA1* mutation. Furthermore, *BRCA1* mutation carriers have significantly lower levels of circulating osteoprotegerin (OPG), the decoy receptor for RANK-ligand (RANKL) and thus endogenous inhibitor of RANK signaling. OPG levels may be associated with the risk of disease, suggesting a role of this protein as a potential biomarker of breast cancer risk. This may improve upon current risk prediction models, stratifying women at the highest risk of developing the disease, and further identify those who may be targets for anti-RANKL chemoprevention. Collectively, the evidence supports therapeutic inhibition of the RANK pathway for the primary prevention of *BRCA1*-associated breast cancer, which may generate unique prevention strategies (without prophylactic surgery) and enhance quality of life.

## Clinical management of women with a *BRCA1* mutation

Women who inherit a pathogenic germline mutation in the *BRCA1* gene face a very high lifetime risk of developing breast cancer, estimated at 72% by age 80 compared to 11% among women in the general population [1, 2]. Current management of these women is limited to either preventive surgery (i.e., prophylactic bilateral mastectomy) or enhanced screening with MRI imaging and mammography [3, 4]. The goal of screening is early detection and whether this modality is a viable alternative to mastectomy has not been established as there are no studies that have compared mortality with MRI screening vs. preventive surgery specifically in this high-risk population. Chemoprevention with selective estrogen receptor modulators such as tamoxifen or aromatase inhibitors is also an option; however, this recommendation is based on data stemming from studies conducted among predominantly non-carriers and there have been no large-scale studies evaluating the effectiveness in the primary prevention of *BRCA1*-associated disease.

In an international study, Metcalfe and colleagues reported that among 6226 healthy women with a *BRCA* mutation, 80% were having regular breast screening, 28% had a preventive mastectomy and only 5% took tamoxifen [5]. This suggests that the rates of prophylactic mastectomy remain low and that the majority of *BRCA1* mutation carriers opt for intensified screening instead [5–7]. Importantly, *BRCA1* mutation carriers have strongly expressed their preference for breast cancer risk reduction and desire a novel prevention drug that is currently not available [8]. Given that the *BRCA1* gene was identified over 20 years ago, that preventive mastectomy remains the gold standard, and that mutation carriers have strong preferences for chemoprevention, it is timely that an effective breast cancer risk reduction option be identified [9, 10].

In this work, we will review the existing experimental and epidemiological evidence implicating dysregulation of the receptor activator of nuclear factor-κB (RANK) signaling pathway in the predisposition to breast cancer among women with an inherited *BRCA1* mutation. An emerging body of data suggests inhibition of RANK as a potential target for prevention in this high-risk population. Furthermore, we (and others) have hypothesized that quantification of circulating osteoprotegerin (OPG), the decoy receptor of RANK-ligand (RANKL), may serve as a potential biomarker of *BRCA1*-associated breast cancer risk that may not only improve upon current risk prediction models, stratifying women at the highest risk of developing the disease, but may further identify those who may be targets for anti-RANKL chemoprevention.

## Emerging role of the RANK signaling pathway in the pathogenesis of *BRCA1*-associated breast cancer

RANK, RANKL, and, OPG are members of the tumor necrosis factor (TNF) and TNF receptor superfamily [11–13]. RANKL can bind to and activate RANK signaling whereas OPG acts as a soluble decoy receptor that binds to RANKL thereby antagonizing RANK/RANKL-mediated signaling [12–14]. The RANK pathway was originally identified as an essential regulator of bone resorption and remodeling [15] but is now known to be involved in physiological and pathological roles beyond bone remodeling, including mammary gland development and tumorigenesis [16–19].

Seminal studies have shown that progesterone-mediated upregulation of the RANK signaling pathway is involved in mammary epithelial proliferation and stem cell expansion and that it also drives tumorigenesis in *Brca1* deficient mice [17–21]. An earlier study by Poole et al. showed that inhibition of progesterone signaling with the progesterone antagonist mifepristone prevented mammary tumorigenesis in *Brca1*-mutant mice [22]. In 2016, two key preclinical studies demonstrated that the inhibition of progesterone-mediated RANK signaling with pharmacological or genetic inactivation suppressed mammary tumor formation in experimental models [23, 24]. Upon examination of mammary tissue in a *Brca1* knockout mouse model, Nolan et al. identified a highly proliferative subset of luminal progenitor cells comprising a larger proportion of the total in *BRCA1*-mutant vs. wildtype mammary tissue [23]. In human mammary tissue specimens, RANK expression was confined to luminal progenitor cells which had a transcriptional signature similar to that of basal-like breast cancers [23]. Furthermore, the authors demonstrated a reduction of progesterone-induced proliferation when RANKL signaling was inhibited with denosumab (a monoclonal antibody to RANKL that is widely used to treat osteoporosis and to prevent skeletal events in breast cancer patients with metastases [25–27]) in a 3D-organoid model derived from mammary cells with a mutation in *BRCA1* and breast biopsies from *BRCA1* mutation carriers [23]. When recombinant OPG (OPG-Fc), a RANKL inhibitor like denosumab, was used for inhibition of RANKL in mice with a *Brca1* mutation, the authors also observed reduced mammary tumor growth [23].

In the same year, Sigl et al. demonstrated that genetic inactivation of RANK signaling in mammary epithelial cells reduced the proliferation of mammary progenitor cells and substantially delayed the onset of mammary tumorigenesis in *Brca1/p53* mutant mice [24]. Remarkably, about 25% of *Rank/Brca1/p53* triple-mutant mice never developed any breast tumors by around day 400 after birth, whereas all *Brca1/p53* double-mutant mice developed tumors by around day 200 [24]. Additionally, the authors demonstrated that pharmacological inhibition of RANK signaling with RANK-Fc, which is similar to OPG-Fc, inhibited the development of pre-neoplastic mammary gland lesions in *Brca1/p53* transgenic mice [24]. Sigl and colleagues also showed the effectiveness of RANKL blockade in human mammary progenitor activity using tissues of *BRCA1* mutation carriers [24]. They reported a significant decrease in vitro clonogenic capacity of progenitor cells after denosumab treatment, highlighting the positive effects of RANKL inhibition to prevent breast cancer [24]. In summary, there is strong evidence indicating the essential role of progesterone-mediated RANK signaling in the expansion of a population of RANK^+^ luminal progenitors which are the likely cells of origin for *BRCA1*-associated basal-like breast cancers [28].

Along the same lines, evidence from epidemiological studies also supports progesterone-mediated upregulation of the RANK pathway in the predisposition to breast cancer among women with a *BRCA1* mutation. In a study by Widschwendter and colleagues, the authors compared circulating levels of sex hormones (i.e., progesterone and estrogen) as well as OPG and soluble RANKL (sRANKL) in *BRCA* mutation carriers (*n* = 391) vs. non-carriers (*n* = 782). They observed 33% higher (*p* = 0.007) levels of luteal phase serum estrogen and 121% higher (*p* = 0.00037) levels of progesterone among *BRCA* mutation carriers, particularly in *BRCA1* mutation carriers, compared with non-carriers across the menstrual cycle [29]. These data support those inherent aberrancies in the levels of sex hormones found in *BRCA* mutation carriers may be associated with the elevated risk of breast cancer. Moreover, serum OPG levels were inversely associated with luteal phase progesterone levels, particularly among *BRCA1/2* mutation carriers (rho = − 0.216; *p* = 0.002) vs. controls (rho = − 0.098; *p* = 0.06) [30]. In macaques, administration of combined estrogen and progestin hormone replacement therapy (HRT), but not estrogen-only HRT, was associated with significantly lower levels of OPG in both breast and serum compared to the control animals not exposed to sex hormones [30]. Lower levels of serum OPG were also associated with increased mammary epithelial proliferation in these macaques (rho = − 0.545, *p* < 0.001), and increased (*p* = 0.01) levels of OPG were observed in postmenopause [30]. However, RANKL upregulation in mammary tissue samples in response to combination HRT was not reflected in the circulation [30]. These data support circulating OPG, but not necessarily RANKL, as a potential marker of local changes in RANK signaling at the breast tissue level and possibly breast cancer risk (see **Section III** below).

Findings from clinical trials have also supported the role of progesterone (rather than estrogen) signaling in development of breast cancer. Most notable are data from the Women’s Health Initiative, randomized trials of HRT, which found a positive correlation between combined estrogen-progestin therapy and breast cancer risk (hazard ratio, HR = 1.55; 95% CI 1.41–1.70) [31, 32]. However, no association was found between estrogen-alone HRT and breast cancer risk (HR = 0.77; 95% CI 0.62–0.95) [33]. This central role of progesterone breast cancer development was the conclusion of a recent meta-analysis of the worldwide evidence of HRT and breast cancer risk which summarized among current users, there were definite risks associated with the use of combined therapy vs. estrogen-alone during years 1–4 (estrogen plus progesterone relative risk, RR = 1.60, 95% CI 1.52–1.69; estrogen-only RR = 1.17, 1.10–1.26) [34]. These definite risks increased two-fold during years 5–14 (estrogen plus progesterone RR = 2.08, 2.02–2.15; estrogen-only RR = 1.33, 1.28–1.37) [34]. During years 5–14, the estrogen plus progesterone risks were greater with daily than with less frequent progesterone use (RR = 2.30, 95% CI 2.21–2.40 vs. RR = 1.93, 95% CI 1.84–2.01; heterogeneity *p* < 0.0001). Interestingly, for *BRCA1* mutation carriers, breast cancer risk decreases after menopause when their sex hormones become substantially low [35, 36]. Importantly, in a prospective analysis by our group, we previously showed that combined HRT use following an oophorectomy has been reported to increase the incidence of breast cancer compared to estrogen-alone HRT among 872 *BRCA1* mutation carriers who underwent bilateral oophorectomy [37]. The cumulative incidence among those who took progesterone HRT was 22% vs. 12% with the use of estrogen-alone (*P*-log rank = 0.04). These associations were stronger for women < 45 years at the time of prophylactic oophorectomy. In the same study cohort, we previously showed that oophorectomy was not associated with a reduced risk of breast cancer (primary and contralateral) substantiating less of a role of estrogen in the pathogenesis of *BRCA1*-associated breast cancer development [38]. Collectively, underlying mechanisms to describe the increased risk of breast cancer likely involve dysregulation of the progesterone-mediated RANK signaling.

## Circulating levels of OPG as a potential marker of breast cancer risk: evidence from the general population and *BRCA1* mutation carriers

Given the considerable experimental and preclinical data implicating RANK signaling in mammary tumorigenesis, there has been increasing interest in the quantification of either circulating sRANKL, OPG, or even the sRANKL/OPG ratio as potential biomarkers of cancer risk. Table 1 summarizes the key characteristics and findings of the five epidemiological studies that have evaluated the relationship between circulating OPG, sRANKL, or OPG/RANKL ratio and breast cancer risk in the general population. Although based on very few studies published to date, these limited data suggest the potential utility of these biomarkers for disease risk prediction.

**Table 1:** Overview of studies assessing the relationship between circulating concentrations of sRANKL or OPG and risk of breast cancer among healthy women without a *BRCA* mutation [28]

Author, Year	Study Source, Study Design, Sample Size	Sample Type	Study Aims	Follow-up	Population Size and Number of Cases	Results
Vik et al., 2015 [39]	Tromsø Study Prospective cohort *n* = ﻿3174 women (range 25–85 years)	Serum	To investigate the association between OPG and risk of breast cancer incident cancer in women	Median: 13.5 years	76 incident breast cancers	RR total upper vs. lower tertile RR = 0.54; 95% CI 0.28–1.06; p_trend_=0.07) RR > 60 years upper vs. lower tertile RR = 1.10; 95% CI 0.49–2.46; p_trend_=0.84) RR < 60 years upper vs. lower tertile RR = 0.24; 95% CI 0.10–0.61; p_trend_ = 0.002)
Fortner et al., 2017 [40]	EPIC cohort Nested case-control *n* = 2008 breast cancer cases matched 1:1 with healthy controls (pre- and post-menopausal women)	Serum	To investigate the association between circulating OPG and breast cancer risk by hormone receptor subtype	Baseline: 1992–2000 End of follow-up: 2003–2006	2008 incident invasive breast cancer cases (1622 ER+, 386 ER–)	Top vs. bottom tertile OPG RR ER– breast cancer = 1.93; 95% CI 1.24–3.02; p_trend_ = 0.03 Top vs. bottom tertile OPG RR ER+ breast cancer = 0.84; 95% CI 0.68–1.04; p_trend_ = 0.18
Kiechl et al., 2017 [41]	UKCTOCS, Bruneck cohorts and SUCCESS trial Case-control *n* = 278 postmenopausal women	Serum	To assess whether serum OPG and RANKL are associated with increased risk of developing breast cancer	Range (cases): 5–24 months Median (controls): 3.24 years	98 breast cancer cases	OR breast cancer in high RANKL/OPG ratio and high progesterone group = 5.33; 95% CI 1.5–25.4; *p* = 0.02
Sarink et al., 2017 [42]	EPIC cohort Nested case-control *n* = 1976 incident invasive breast cancer matched 1:1 with healthy controls (median age at blood collection: 56 years (range 27–77 years))	Serum	To investigate the association between serum sRANKL levels and breast cancer risk by hormone receptor subtype	Baseline: 1992–2000 End of follow-up: 2003–2006	1976 incident invasive breast cancer cases (1598 ER+)	Serum sRANKL associated with ER+ disease (5th vs. 1st quintile RR = 1.28; 95% CI 1.01–1.63; p_trend_ = 0.20) No association between serum sRANKL and ER– disease (5th vs. 1st quintile RR = 0.87; 95% CI 0.53–1.44; p_trend_ = 0.21)
Kotsopoulos et al., 2020 [43]	NHS II Nested case-control study *n* = 297 incident invasive breast cancer (premenopausal women) matched 1:1 with healthy controls (median age at blood collection: 44 years (range 41–47 years))	Plasma	To investigate the association between plasma OPG and breast cancer risk	Baseline: 1989–1990 End of follow-up: 2009	297 incident invasive breast cancer cases	No association between plasma OPG and breast cancer risk (highest vs. lowest quartile OR = 0.78; 95% CI 0.46–1.33; p_trend_ = 0.30)

*Abbreviations*: *CI* Confidence Interval, *EPIC* European Prospective Investigation into Cancer and Nutrition, *ER* estrogen receptor, *OPG* osteoprotegerin, *OR* odd ratios, *NHS II* Nurses’ Health Study II, *RR* relative risk, *sRANKL* soluble receptor activator of nuclear factor κB ligand, *UKCTOCS* UK Collaborative Trial of Ovarian Cancer Screening

Briefly, a prospective study conducted by Vik et al. showed when stratified by age and sex, a significant inverse relationship between serum OPG and breast cancer risk in women under 60 years of age, but not in women above 60 years of age after adjustment, although the sample size was relatively small (upper vs. lower tertile RR = 0.24; 95% CI 0.10–0.61; p_trend_ = 0.002) [39]. While the authors observed a linear relationship between the overall cancer-related mortality and serum OPG (RR of cancer-related mortality increased by 25% per 1 standard deviation increase in serum OPG, RR 63% higher in upper vs. lower tertile), only 7 out of 6279 cases were related to breast cancer [39]. Interestingly, Vik et al. observed that high serum OPG levels were associated with higher risk of gastrointestinal cancer (HR = 1.79; 95% CI 1.19–2.67) [39].

Similarly, Fortner et al. conducted a case-control study nested in the European Prospective Investigation into Cancer and Nutrition (EPIC) cohort to investigate the association between circulating OPG levels and breast cancer risk by hormone receptor type and matched breast cancer cases with healthy controls [40]. The authors found that the high level of circulating OPG was a significant risk factor for estrogen receptor (ER)-negative breast cancer development (top vs. bottom tertile OPG RR ER-negative breast cancer = 1.93; 95% CI 1.24–3.02; p_trend_ = 0.03) but not for ER-positive breast cancer development (top vs. bottom tertile OPG RR ER-positive breast cancer = 0.84; 95% CI 0.68–1.04; p_trend_ = 0.18) [40].

In the same year, Kiechl et al. published a case-control study that assessed whether serum OPG and RANKL levels were associated with the risk of developing breast cancer for postmenopausal women without a *BRCA* mutation germline [41]. In this study, 98 women developed breast cancer and women with high ratio of RANKL/OPG serum levels and high progesterone exhibited a 5.33-fold higher risk of developing breast cancer (OR breast cancer in high RANKL/OPG ratio and high progesterone group = 5.33; 95% CI 1.5–25.4; *p* = 0.02) [28, 41]. In a subsequent analysis of sRANKL in the EPIC cohort, Sarink et al. observed an inverse association of serum sRANKL/OPG ratio with ER-negative breast cancer (5th vs. 1st quintile RR = 0.60; 95% CI 0.31–1.14; p_trend_ = 0.03). The authors also observed a positive association between serum sRANKL and ER-positive breast cancer (5th vs. 1st quintile RR = 1.28; 95% CI 1.01–1.63; p_trend_ = 0.20) but not ER-negative disease [42]. Additional studies are necessary to explain why these associations differ by breast cancer subtype.

Conversely, in a recent study conducted by Kotsopoulos et al., the authors observed no substantial evidence of an association between plasma OPG and breast cancer risk among premenopausal women in a case-control analysis nested within the Nurses’ Health Study II; however, the authors observed a suggestive inverse trend and their analyses may have been underpowered (highest vs. lowest quartile OR = 0.78; 95% CI 0.46–1.33; p_trend_ = 0.30) [43].

The three studies that have evaluated the relationship of OPG and/or sRANKL with breast cancer risk specifically among women with a *BRCA1* or *BRCA2* mutation are summarized in Table 2. In the previously described study by Widschwendter et al., the authors reported significantly lower serum OPG concentrations among premenopausal *BRCA* mutation carriers, particularly in *BRCA1* mutation carriers (*p* = 0.018), compared with non-carriers [30]. They did not measure risk associated with levels of OPG but did find that higher levels of circulating OPG were associated with lower risk pathogenic germline mutations in *BRCA1/2* mutation carriers (beta = − 0.058; 95% CI − 0.020, − 0.096; *p* = 0.003) [30]. In a prospective cohort study by Odén et al., the authors showed that high vs. low plasma OPG was associated with risk of developing breast cancer among *BRCA1* and *BRCA2* mutation carriers (*n* = 206; HR = 0.25; 95% CI 0.08–0.78; *p* = 0.02) [44]. However, there was no association between plasma RANKL and breast cancer risk when stratified into high vs. low plasma RANKL levels (HR = 1.06; 95% CI 0.34–3.28; *p* = 0.86) [45]. Although limited, the evidence points towards a role of dysregulated RANK signaling in the development of *BRCA*-associated breast cancer and thus the potential for chemoprevention with a RANKL inhibitor such as denosumab [30]. Furthermore, this suggests that the integration of circulating OPG levels (or the sRANKL/OPG ratio) into risk prediction models may have the potential to identify women who are at the highest risk of developing breast cancer.

**Table 2:** Summary of studies assessing the relationship between circulating concentrations of sRANKL and/or OPG and risk of breast cancer among *BRCA1* and *BRCA2* mutation carriers [28]

Author, Year	Study Source, Study Design, Sample Size	Study Aims	Follow-up	Population Size and Number of Cases	Results
Widschwendter et al., 2015 [30]	UKFOCSS Cross-sectional *n* = 391 *BRCA1/2* mutation carriers and 782 healthy controls (> 35 years)	To evaluate the relationship between the *BRCA1/2* mutation and levels of sRANKL and OPG To assess the relationship between reported breast cancer risk associated with the nucleotide position of the *BRCA1/2* germline mutation and serum OPG concentrations	N/A	N/A	Lower serum OPG and sRANKL levels in *BRCA1/2* mutation carriers vs. healthy controls Germline *BRCA1/2* mutation locations known to confer an increased risk of breast cancer were associated with lower OPG levels
Odén et al., 2016 [44]	Risk Factor Analysis of Hereditary Breast and Ovarian Cancer Prospective cohort *n* = 206 *BRCA1/2* mutation carriers between 18 and 70 years	To assess whether plasma OPG levels contribute to breast cancer risk in *BRCA1/2* mutation carriers	Mean: 6.5 years (0.1–18.8 years)	18 incident breast cancer cases	High vs. low OPG HR breast cancer = 0.25 (95% CI 0.08–0.78), *p* = 0.02
Zaman et al., 2019 [45]	Risk Factor Analysis of Hereditary Breast and Ovarian Cancer Prospective cohort *n* = 184 *BRCA1/2* mutation carriers between 18 and 80 years	To investigate the association between plasma RANKL levels and breast cancer risk in *BRCA1/2* mutation carriers	Mean: 6.3 years (0.02–19.24 years)	15 incident breast cancer cases	High vs. low RANKL HR breast cancer = 1.06 (95% CI 0.34–3.28), *p* = 0.86

*Abbreviations*: *CI* Confidence Interval, *HR* hazard ratio, *OPG* osteoprotegerin, *sRANKL* soluble receptor activator of nuclear factor κB ligand, *UKFOCSS* UK Familial Ovarian Cancer Screening Study

## Non-genetic and genetic regulators of OPG and RANKL expression

Several studies have reported upon or evaluated how various hormonal factors, cytokines, growth factors may influence mRNA expression or protein levels of OPG and sRANKL. For example, estrogen, IL1α, TNFα, and TGFß upregulate OPG expression [46–50] whereas parathyroid hormone and parathyroid hormone-related prostaglandin E2 and glucocorticoids protein downregulate OPG expression [51–60]. The role of vitamin D3, cytokines and growth factors is less consistent and studies reported mixed results [55, 61–70]. Overall, OPG expression is upregulated by many suppressors of osteoclast differentiation (i.e., transforming growth factor β, estrogen) and downregulated by some pro-resorptive agents (i.e., parathyroid hormone, prostaglandins) [28, 71]. Most studies report reciprocal regulation of OPG and RANKL, where factors that downregulate expression of OPG will upregulate expression of RANKL, and vice-versa [28, 71]. This reciprocal regulation modulates pro-resorptive compounds to stimulate resorption via RANKL induction while inhibiting effects of OPG [28]. Estrogen, TGFß, and interferon-ɣ downregulate RANKL expression, whereas vitamin D3, parathyroid hormone, and parathyroid hormone-related prostaglandin E2, IL1α, IL1ß, IL11, IL17, TNFα, prostaglandin E2, and glucocorticoids upregulate RANKL expression [46, 47, 51–54, 57, 59, 64–67, 69]. Yet, there are some conflicting findings from one publication to another.

Epidemiological studies have also evaluated correlates of OPG. Notable, numerous studies have shown a strong positive correlation between OPG and age in women [40, 72–75]. The onset of menopause is highly related to age and decreased estrogen levels are one of the contributors to rapid bone loss. It is hypothesized that this decline in estrogen levels can decrease OPG production in osteoblasts and promote bone resorption [47, 48, 76]. Conversely, Sarink et al. found that OPG concentrations in healthy pre- and post-menopausal women in the general population are minimally impacted by hormonal lifestyle factors [77]. Other studies also found mixed, and often weak, associations with OPG levels in healthy women with circulating endogenous sex hormones, such as estradiol, menopause, and HRT use [40, 72, 78, 79].

For high-risk women, it appears that an inherited *BRCA1* and *BRCA2* are associated with significantly lower circulating OPG levels. As previously described, a *BRCA1* or *BRCA2* mutation is associated with decreased circulating serum OPG expression. Beyond the *BRCA* genes, a few studies investigated genetic factors associated with circulating OPG levels [80–84]. Recently, Kwan et al. conducted a meta-analysis of five genome-wide association studies comprising individuals from European and Asian origin (*n* = 10,336) [82]. The authors discovered two significant genome-wide significant loci (8q23-q24.1, located > 100 kb upstream of the gene that encodes OPG; and 17q11.2) and one locus (14q21.2) with near genome-wide significance associated with circulating OPG levels (mostly OPG serum levels) [82]. They estimated that over half of the heritability of age-adjusted OPG levels could be explained by all SNPs examined in their study [82]. Interestingly, Kwan et al. evaluated the association between a single nulceotide polymorphism (SNP) rs875525 in the *ANKH* gene and OPG levels however it did not reach genome-wide significance in their meta-analyses [82]. In contrast, Vistoropsky et al. found a significant association between a SNP rs875525 in the *ANKH* gene and plasma OPG levels from their family-based association study (*n* = 556) [84]. Collectively, the current literature demonstrates inconsistent findings regarding genetic regulators of OPG expression, which emphasizes the need for additional work on fine-mapping these regions and identifying other casual variants using a large sample size [82].

Overall, our current understanding of factors regulating OPG expression is limited and the conflicting findings among different studies make it challenging to come to a consensus. This is further confounded by the complexity of the RANK signaling pathway that can be regulated by the interaction of various factors and as the changes in serum OPG levels may not reflect the tissue of interest [85]. Nonetheless, OPG is a promising biomarker for *BRCA1*-associated breast cancer risk due to the association between *BRCA1* mutation and OPG levels [30].

## Conclusion and future directions

Here we have presented a brief overview of the experimental and epidemiologic evidence suggesting that dysregulation of the progesterone-mediated RANK signaling pathway plays a critical role in the pathogenesis of *BRCA1*-associated breast cancer. Based on observational data, *BRCA1* mutation carriers have significantly lower concentrations of circulating OPG [30], and OPG levels may be associated with the risk of disease. This suggests the potential role of integrating OPG levels (or the sRANKL/OPG ratio) to improve upon cancer risk prediction models. Furthermore, the preclinical and experimental data support therapeutic inhibition of the RANK pathway for the primary prevention of *BRCA1*-associated breast cancer [23, 24].

Randomized controlled trials are needed to evaluate the clinical effectiveness of denosumab for *BRCA1*-associated breast cancer prevention in these high-risk women. Whether it is effective for the prevention of ovarian cancer or *BRCA2* mutation carriers is not known. It is of interest to see whether this pathway will similarly apply to ovarian cancer. Ultimately, denosumab may provide a necessary, non-surgical, and effective risk reduction intervention for a population at substantial risk for developing breast (and potentially ovarian) cancer. Although a risk-reducing mastectomy is a highly effective procedure for women with a *BRCA1* mutation, the low uptake and high opt-out rate for these women for screening instead underlies the need to develop new, non-invasive preventative agents and strategies for managing *BRCA1*-associated breast cancer risk.

## Data Availability

Not applicable.

## Abbreviations

BRCA1: Breast cancer type 1 susceptibility protein
BRCA2: Breast cancer type 2 susceptibility protein
CI: Confidence interval
EPIC: The European Prospective Investigation into Cancer and Nutrition
ER: Estrogen receptor
HR: Hazard ratio
HRT: Hormone replacement therapy
IL: Interleukin
OPG: Osteoprotegerin
OR: Odds ratio
RANK: Receptor activator of nuclear factor κB
RANKL: Receptor activator of nuclear factor κB ligand
RR: Relative risk
SNP: Single nucleotide polymorphism
sRANKL: Soluble receptor activator of nuclear factor κB ligand
TGF: Transforming growth factor
TNF: Tumor necrosis factor
